# CRISPR single base editing, neuronal disease modelling and functional genomics for genetic variant analysis: pipeline validation using Kleefstra syndrome EHMT1 haploinsufficiency

**DOI:** 10.1186/s13287-022-02740-3

**Published:** 2022-02-09

**Authors:** Vanessa S. Fear, Catherine A. Forbes, Denise Anderson, Sebastian Rauschert, Genevieve Syn, Nicole Shaw, Sarra Jamieson, Michelle Ward, Gareth Baynam, Timo Lassmann

**Affiliations:** 1 Translational Genetics, Precision Health, Telethon Kids Institute, Northern Entrance, Perth Children’s Hospital, 15 Hospital Avenue, Nedlands, WA 6009 Australia; 2 Computational Biology, Precision Health, Telethon Kids Institute, Perth Children’s Hospital, Nedlands, WA 6009 Australia; 3 Western Australian Register of Developmental Anomalies, King Edward Memorial Hospital, Subiaco, WA 6008 Australia; 4 Undiagnosed Diseases Program, Genetic Services of WA, Subiaco, Australia

**Keywords:** Rare genetic diseases, Translational genetics, Kleefstra syndrome, CRISPR SNV editing, Variant of uncertain significance, Inducible pluripotent stem cells, Functional genomics

## Abstract

**Background:**

Over 400 million people worldwide are living with a rare disease. Next Generation Sequencing (NGS) identifies potential disease causative genetic variants. However, many are identified as variants of uncertain significance (VUS) and require functional laboratory validation to determine pathogenicity, and this creates major diagnostic delays**.**

**Methods:**

In this study we test a rapid genetic variant assessment pipeline using CRISPR homology directed repair to introduce single nucleotide variants into inducible pluripotent stem cells (iPSCs), followed by neuronal disease modelling, and functional genomics on amplicon and RNA sequencing, to determine cellular changes to support patient diagnosis and identify disease mechanism.

**Results:**

As proof-of-principle, we investigated an *EHMT1* (Euchromatin histone methyltransferase 1; EHMT1 c.3430C > T; p.Gln1144*) genetic variant pathogenic for Kleefstra syndrome and determined changes in gene expression during neuronal progenitor cell differentiation. This pipeline rapidly identified Kleefstra syndrome in genetic variant cells compared to healthy cells, and revealed novel findings potentially implicating the key transcription factors REST and SP1 in disease pathogenesis.

**Conclusion:**

The study pipeline is a rapid, robust method for genetic variant assessment that will support rare diseases patient diagnosis. The results also provide valuable information on genome wide perturbations key to disease mechanism that can be targeted for drug treatments.

**Supplementary Information:**

The online version contains supplementary material available at 10.1186/s13287-022-02740-3.

## Background

Rare diseases are collectively prevalent, with over 80% having genetic basis. Currently, Next Generation Sequencing (NGS) has provided a rapid, and ever more affordable tool for the identification of genetic variants in rare disease patients. However, whilst NGS leads to a diagnosis in up to 50% of patients, a large proportion of identified genetic variants are novel and therefore classified as variants of uncertain significance (VUS) meaning the patient cannot receive a diagnosis [1]. These identified VUS require functional validation to determine disease pathogenicity, a process currently performed using functional assays in highly specialised laboratories, that is undertaken in ad hoc fashion. This requirement for functional validation of VUS leads to a significant delay in rare diseases patient diagnosis of  > 5 years, if not decades [2]. Faster and more systematic approaches to genetic variant validation are required. We propose a new and rapid pipeline for VUS assessment using a pipeline of CRISPR single base editing, inducible pluripotent stem cell disease modelling, and functional genomics. To demonstrate this pipeline we assess a known patient genetic variant EHMT1_c.3430C > T (p.Gln1144*) CRISPR edited into human inducible pluripotent stem cells (iPSCs), and determine genome wide changes in neural progenitor cell differentiation by functional genomics.

Kleefstra syndrome is caused by *EHMT1* haploinsufficiency [3] that results in partial or complete loss of EHMT1 expression [4]. *EHMT1* encodes a histone lysine methyltransferase [5], which forms a complex with EHMT2, for regulation of gene expression by histone 3 lysine 9 (H3K9) mono- and di-methylation (H3K9me1 and H3K9me2) [6]. The highly modulatory nature of histone mono-, di- or tri methylation leads to complex transcriptional regulation, based on the specific residues and the pattern of methylation [7, 8]. Kleefstra Syndrome [9, 10] hallmark symptoms include mental retardation, intellectual disability associated with autistic features, limited or absent speech, seizures, hypotonia, microcephaly, and brachycephaly. Other common symptoms are evident in the face (flat face, prognathism, midface hypoplasia and coarse facies), malformed ears, eyes (hypertelorism, upslanting palpebral fissures), and hands (brachydactyly, single transverse palmar creases), amongst others. Structural brain abnormality, congenital heart defects, seizures, high birth weight and/or childhood obesity are also reported.

In this proof-of-principle study we assess a Kleefstra Syndrome patient’s known pathogenic *EHMT1* genetic variant. The patient genetic variant EHMT1_c.3430C > T; p.Gln1144* (herein referred to as *EHMT1*_SNV) was introduced into iPSCs, followed by neuronal cell differentiation. Differentiation of iPSCs to neural progenitor cells was compared between *EHTM1*_WT (wild-type) cells and *EHMT1*_SNV cells using RNA sequencing and transcriptomics analysis. Analysis of differentially expressed genes (DEGs) revealed enrichment of gene sets and transcription factor motifs associated with Kleefstra syndrome, changes in neuronal development, and provided insight into disease mechanisms.

## Methods

### Patient recruitment

The patient recruitment to this study was initiated by a genetic counsellor based at Genome Services Western Australia, followed by written informed consent. The study is in line with the Declaration of Helsinki and the NHMRC National Statement on Ethical Conduct Human Ethics Research. Approved by the Child and Adolescent Health Services, Human Research Ethics Committee, RGS0000000166.

The patient genetic variant was determined with Massively Parallel Sequencing via Trusight One, and a genetic variant detected NM_024757.4(EHMT1): c.[3430C > T];[ =] p.[(Gln1144*)];[( =)] which concluded EHMT1 Haploinsufficiency, which was considered pathogenic for Kleefstra syndrome [3].

### Cell culture

KOLF2-C1 (KOLF2; HipSci) cells were grown in 24-well plates coated with Celladhere Vitronectin XF (STEMcell technologies), maintained in TeSR-E8 media (STEMCELL technologies), and media changed daily. Cells were split with Gentle Cell Dissociation Reagent with 10 µM ROCK Inhibitor (Y-27632, STEMCELL Technologies) for 24 h on re-plating. Cell cryopreservation was in CryoStor CS10 (STEMCELL Technologies). All cultures were grown in a 37 °C humidified CO_2_ (5%) incubator, unless otherwise stated, routinely checked for mycoplasma contamination.

### CRISPR/Cas9 *EHMT1*_SNV transfection and cloning

KOLF2 cells grown to 30–50% confluence, were dissociated with Gentle Cell Dissociation Reagent and 1 × 10^5^ cells in 400 µl mTeSR1 with 10 µM Y-27632 (STEMCELL technologies) aliquoted in 24 well plates. HDR and crRNA were covalently linked with Click Chemistry [10]. In brief, 10 µM EHMT1_HDR, ssDNA (5’AzideN CTCTACCGGACGCGGGACATGGGCTGGGGCGTGCGGTCTCTCTGAGACATTCCTCCTGGAACCTTTGTCTGCGAGTGAGTGAGT3’), and 10 µM crRNA (5’ Dibenzocyclooctyne N-Hydroxysuccinimide, DBCON, sequence: 5’TCCCTGCAGGACATCCC3’, PAM: AGG; EHMT1 NM_024757.2) were incubated at room temperature overnight to create a gDonor molecule [10]. The gRNA was generated in Duplex Buffer (IDT, USA) with 1 µM gDONOR (Integrated DNA Technologies), 1 µM tracrRNA-ATTO550 (Integrated DNA Technologies), heated to 95 °C for 5 min and cooled to RT. CRISPR ribonucleoprotein (RNP complexes) were formed with 63 nM gRNA and 63 nM high-fidelity Cas9 protein (Integrated DNA Technologies) in OPTIMEM with STEM Lipofectamine Cas Plus reagent (Life Technologies) and OPTIMEM. RNPs were transfected into KOLF2 cells with STEM Lipofectamine (Life Technologies), according to the manufacturer’s instruction. Alternatively, cells were transfected on the Neon Transfection system (1400 V, 20 ms, 1 pulse; Invitrogen). Cells were cultured for at least 7 days prior to cell freeze down or genomic DNA extraction using PureLink™ Genomic DNA Mini Kit (Life Technologies). Percentage frequency of HDR to create the *EHMT1*_SNV were determined by Amplicon sequencing. Transfected cells were then single cell cloned by limiting dilution in 96 well plates, replica plated, and DNA lysate prepared [11]. Further rounds of amplicon sequencing on DNA lysate determined clonal cell lines. Positive clones were thawed, re-tested and cell stocks prepared. Schematic of EHMT1 genetic variant introduction illustrated in Additional file 1: Fig. S1a. The *EHMT1*_SNV variant sequence mutalyzer variant description is NM_024757.5 (EHMT1):c.3426_3447delinsTCTCTGAGACATTCCTCCGGGA, with affected transcript NM_024757.5(EHMT1_i001):p.(Gln1144*). The top six off target CRISPR crRNA gene cut sites were confirmed as WT by forward and reverse Sanger sequencing (AGRF, WA; Additional file 2: Table S1. EHMT1 off target).

### Amplicon sequencing

Next-generation amplicon sequencing was carried out on the MiniSeq Sequencing System (Illumina^©^). In brief, a 267 bp *EHMT1*_Ter site PCR product was amplified, from gDNA or DNA lysates, with *EHMT1* pAMPF3 (5’ACACTCTTTCCCTACACGACGCTCTTCCGATCTCTCTCTATTTTTCAGGGCAAGG3’) and *EHMT1* pAMPR2 (5’GTGACTGGAGTTCAGACGTGTGCTCTTCCGATCTACAGCACGAGCTTGGTTCTC3’), using the CRES-Seq [11] for 150 bp, paired end, > 10,000 reads (MiniSeq, Illumina, Australia) and reads aligned to the HDR or WT amplicon with CRISPResso2 software [12].

Cells were plated at 20 cells/well and wells positive for the *EHMT1*_SNV genetic variant determined with *EHMT1* amplicon sequencing [11]. Cells in positive wells were then single cell cloned followed by *EHMT1* amplicon sequencing [11] to identify three heterozygous EHMT1_SNV iPSC clones (*EHMT1_*WT/SNV; *EHMT1*_SNV_16, *EHMT1*_SNV_17, *EHMT1*_SNV_29) and three matched *EHMT1* wild-type clones (*EHMT1*_WT_100, *EHMT1*_WT_102, *EHMT1*_WT_103).

### Neural progenitor cell differentiation

KOLF2 iPSCs*,* genetic variant and normal, were stimulated to differentiate into neural progenitor cells with STEMdiff SMADi Neural Induction Kit (STEMCELL, Vic, Australia). At day 0, and 24 of neural cell differentiation 5 × 10^5^ cells were collected, LIVEDEAD ef780 stained for 10 min (eBioscience, US), fix/permeabilized according to Transcription factor staining buffer set (eBioscience, US) and stained for stem cell expression marker (OCT3/4), and neuronal cell marker (PAX6). Samples were collected using an LSRII X20 flow cytometer (BD, Biosciences), and analysed with FlowJo software (TreeStar Inc, Ashlan, OR, USA). Gating strategy was total cells, with subsequent gating on live cells and then singles, prior to determination of percentage frequency protein marker expression. Statistical analysis of data was with one-way ANOVA and Bonferroni’s correction for multiple testing performed using GraphPad Prism Version 8 software (GraphPad Software Inc., La Jolla, CA, USA). In addition, cells were collected for RNA extraction (RNeasy Minikit, with DNase on column treatment; Qiagen).

### EHMT1 Western blot and immunohistochemistry

Proteins were extracted from cells lysed with Pierce’s co-IP protein lysis buffer (ThermoFisher Scientific), quantified (Direct-Detect® Infrared Spectrometer, Merck Millipore), electrophoresed on NuPAGE Bis–Tris 4–12% protein gels (Life Technologies) and blotted onto Polyvinylidene fluoride (PVDF) membranes (Life Technologies). Membranes were blocked with Intercept® (TBS) Blocking Buffer (LI-COR® Biosciences) at 4ºC overnight, stained with rabbit anti-human EHMT1 (1:1000, NBP2-57,166, Novus Biologicals, USA), and/or b-Actin (1:5000; MA5-15,729; Life Technologies, Australia), with secondary stain with goat anti-rabbit IRDye® 800CW (1:10,000; LI-COR® Biosciences) or goat anti-mouse IRDye® 680RD (1:10,000; LI-COR® Biosciences) and imaged on the Odyssey® Infrared Imaging System (LI-COR® Biosciences).

*EHMT1*_WT and *EHMT1*_SNV iPSCs were cultured on Matrigel-coated chamber slides (ibidi). One day later, cells were fixed with 3.7% formaldehyde (Sigma-Aldrich) for 20 min at room temperature. Subsequently, cells were permeabilised for 15 min with 0.1% Triton-X-100 (Sigma-Aldrich) and blocked with Intercept® Blocking Buffer (LI-COR) for 1 h at room temperature. Cells were incubated overnight at 4 °C with rabbit polyclonal anti-EHMT1 antibody (1:200; Novus Biologicals), washed with 0.05% Tween-20 (Sigma-Aldrich) and incubated with Alexa-Fluor 488-conjugated anti-rabbit antibody (1:1000; Invitrogen) for 1 h at room temperature. After washing cell nuclei were stained using NucBlue stain (Invitrogen) according to the manufacturer’s instructions. Antibody staining was visualised using a Nikon Eclipse TS2R inverted fluorescence microscope, images captured using a monochrome DS-Qi2 camera (Nikon) and immunofluorescence images processed using NIS-Elements software (v.5.21.00) and Adobe Photoshop (v.22.3.1) to add colour and merge channels. A negative control containing no primary antibody was used to set the background fluorescence.

### RNA sequencing

RNA integrity was determined on the bioanalyser (Australian Genomics Research Facility (AGRF, Perth, Western Australia). RNA sequencing was performed according to SureSelect Strand-Specific RNA Library Preparation for Illumina Multiplexed Sequencing, paired-end, 100 bp, 30 M read RNA sequencing on the NOVAseq 6000 platform (Illumina, USA) at Genomics WA (Perth. Australia).

### Preprocessing, exploratory data analysis and differential analysis

#### Data pre-processing

Raw sequencing reads were processed using the ENCODE ‘rna-seq-pipeline’ (https://github.com/ENCODE-DCC/rna-seq-pipeline), via the Cromwell wrapper software ‘caper’ (https://github.com/ENCODE-DCC/caper). Within the pipeline, reads were aligned to GRCh38 using the STAR aligner (v2.5.1b) [13] and known transcripts were quantified using Kallisto (v0.44.0) [14]. We created gene and transcript expression tables for downstream analysis with *DESeq2, limma* and *DRIMSeq* [15–17] using the package *tximport* [18].

#### Exploratory data analysis

Gene counts were read into R 4.0.2 (https://www.R-project.org/) using the *DESeq2* package function DESeqDataSetFromTximport() [15]. We filtered the counts to retain genes with a count of 3 or more and normalised the data using the variance stabilising transformation [19]. The plotPCA() function was used to produce a principal component analysis plot based on the top 500 variable genes and no outlying samples were observed.

Differential gene and transcript expression analysis. We used *limma* to fit linear models to test for differentially expressed genes and transcripts to address the following hypotheses: (i) Expression differs when comparing *EHMT1*_SNV versus *EHMT1*_WT in NPCs; (ii) Expression differs when comparing *EHMT1*_SNV versus *EHMT1*_WT in iPSCs; (iii) Expression differs during differentiation from iPSCs to NPCs for *EHMT1*_SNV; (iv) Expression differs during differentiation from iPSCs to NPCs for *EHMT1*_WT; and, (v) Changes in expression during differentiation from iPSCs to NPCs differs when comparing *EHMT1*_SNV to *EHMT1*_WT. Genes were filtered using the filterByExpr() function accounting for the number of samples in each group and limma-trend models were fit to normalised log_2_ counts per million transformed data, calculated using the cpm() function with a prior count of 3. We determined quality weights for each sample using the arrayWeights() function and adjusted for this in the models. We also adjusted for pairing of samples from the same clone using the duplicateCorrelation() function. Genes and transcripts with Benjamini-Hochberg [20] corrected *p*-values less than 0.05 and absolute fold-changes of at least 2 were deemed to be differentially expressed (DE).

#### Functional enrichment analysis

We performed functional gene set enrichment analysis on the differentially expressed genes using Enrichr [21, 22] and we used Benjamini–Hochberg corrected *p*-values when determining significance. We also performed Gene Set Enrichment Analysis (GSEA) [23, 24] on all genes using the moderated t-statistic from the differential expression analysis for ranking and tested for enrichment in the Molecular Signatures Database v7.1 (MSigDB) hallmark gene sets [25] using a false discovery rate less than 0.05 to determine significance.

### Upstream regulatory analysis

Analysis was performed to find enriched DNA motifs in regions upstream of the identified differentially expressed genes. For this analysis, the 500 base pairs (bp) upstream of the start site of the differentially extracted genes were extracted from *GRCh38*, using the *bedtools* function *getfasta* [26]. The resulting two stranded base sequences were analysed with the *MEME* software (meme-suite.org) to identify motifs upstream of the genes [27]. Motif length search space was between 3 and 20, with a default E-value threshold of 0.05.

Discriminative motif discovery analysis was performed between the over and underexpressed genes, the overexpressed and background genes, and the underexpressed and background genes, using the *DREME* software [28]. The background genes are defined as genes that passed the filtering step in the limma DE analysis and were not differentially expressed in any of the statistical comparisons. *TomTom* was used [29] to compare the potential motifs identified by *MEME* and *DREME* to the *JASPAR 2018 vertebrate redundant* database [30] of known motifs.

*AME* software was used to identify overrepresented known motifs [31]. The sequences 500 bp upstream from the significantly over and underexpressed genes, as well as all differentially expressed genes were contrasted against the background sequences, with the *JASPAR 2018 vertebrate redundant* database as search database [31]. Significant transcription factors were identified via Fisher's exact test, optimized over motif scores (significance threshold ≤ 0.05).

## Results

### Kleefstra syndrome EHMT1 patient genetic variant

A Kleefstra Syndrome patient was identified to have the genetic variant *EHMT1*_c.3430C > T (p.Gln1144*). The patient’s phenotype was characterised using the Human Phenotype Ontology terms as detailed in Additional file 2: Table S2.

**Fig. 1:**
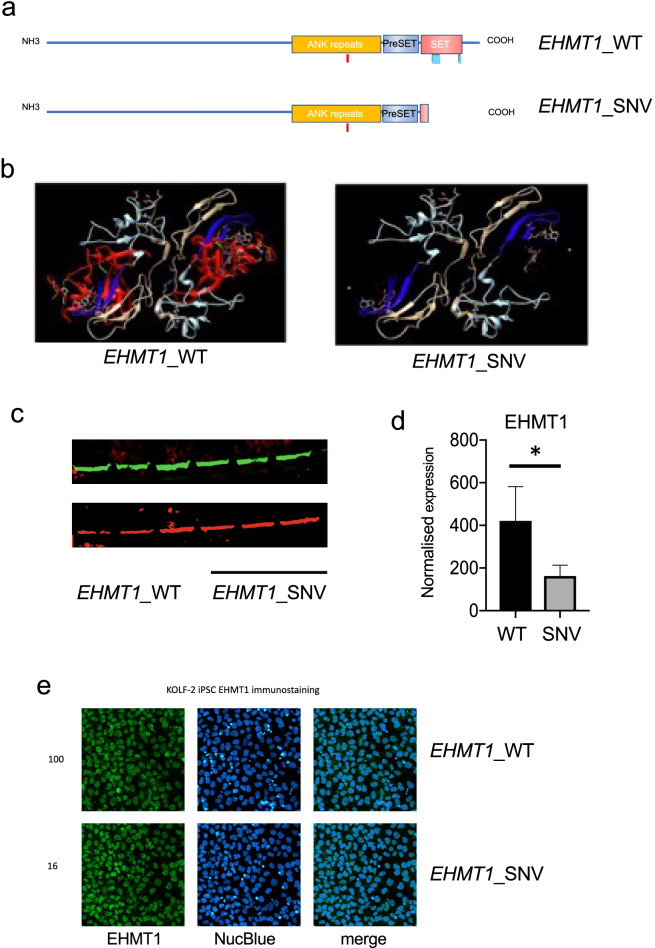
Changes in EHMT1 protein expression in *EHMT1*_SNV cells. **a** Two-dimensional protein structure of EHMT1_WT and EHMT1_SNV with Ankyrin repeat domain (ANK Repeats), PreSET domain, and SET domain. **b** Three dimensional structure of EHMT1_WT and EHMT1_SNV indicating Pre-SET domain (light blue), and the SET domain with methyl transferase activity in blue and red, In the EHMT1_WT protein the red colouring indicates the region absent in the EHMT1_pGln1144* mutant protein., and the green indicates the truncated protein stop codon. **c** Western blot of EHMT1 and b-actin protein expression in *EHMT1*_WT cells and *EHMT1*_SNV cells. **d** Bar graph indicates EHMT1 protein expression normalized to b-actin control (paired t-test, one-tailed, *p < 0.05). **e**, Immunohistochemistry staining of EHMT1 and NucBlue, and merged image, in *EHMT1*_WT and *EHMT1*_SNV iPSCs, white bar indicates 50micron

Euchromatic lysine histone methyltransferase proteins are characterised by the presence of SET/pre-SET domains that are responsible for methyltransferase activity [32–34]. The EHMT1 protein also has 6 ankyrin repeat domains that provide binding specificity for H3K9me1 and H3K9me2 to enable chromatin reading. Two- and three-dimensional protein structures for the EHMT1_WT and EHMT1_SNV protein are illustrated (Fig. 1a, b). The 2D protein structure clearly indicates EHMT1_SNV loss of the SET binding domain necessary for protein–protein interaction, and loss of the two histone H3 binding sites, whereas the PreSET domain and histone 3 lysine 9 (H3K9) binding site remain intact. To visualise the effects of the variant on protein structure, 3D models of EHMT1 wild-type and mutant proteins were superimposed onto the protein data bank (PDB) entry: ‘5TUZ structure of human GLP SET-domain (EHMT1) in complex with inhibitor MS0124 (https://doi.org/10.2210/pdb5TUZ/pdb)’. This highlighted changes in functional domains that may affect function for the variant EHMT1_SNV protein.

The *EHMT1*_SNV variant was introduced into KOLF2 iPSCs using CRISPR_HDR. After transfection polyclonal *EHMT1* gene modifications were determined by amplicon sequencing [12]. A HDR rate of 1.1 ± 0.11% was achieved for the introduction of the patient EHMT1 genetic variant in KOLF2 iPSC transfected cells, with a NHEJ frequency of 26.47 ± 2.83%. Our amplicon sequencing and cloning method allowed the rapid identification of *EHMT1* alleles and selection of cell clones that were heterozygous for the *EHMT1*_SNV genetic variant.

Next, we determined the protein and gene expression level of EHMT1 for the *EHMT1*_WT and *EHMT1*_SNV cells. EHMT1 expression may be reduced by degradation of the truncated protein, or degradation of RNA due to the premature STOP codon [35, 36]. The expression of EHMT1 protein was significantly reduced in the *EHMT1*_SNV iPSCs compared to the *EHMT1*_WT iPSCs (Fig. 1c, d), and the truncated form of the protein was not detected. EHMT1 protein expression was localised to the nucleus on immunohistochemistry analysis of *EHMT1*_SNV and *EHMT1*_WT cells (data not shown). The RNA-seq *EHMT1* gene expression for *EHMT1*_SNV compared to *EHMT1*_WT iPSCs also showed reduced expression, however, did not meet our *p*-value and fold change threshold for DEGs (log_2_FC =  − 0.49, *p* = 0.23).

This data indicates a reduction in EHMT1 protein expression in *EHMT1*_SNV cells compared to the *EHMT1*_WT cells that may affect cell function and differentiation.

### *EHMT1*_WT and *EHMT1*_SNV iPSCs differentiate into neural progenitor cells

**Fig. 2:**
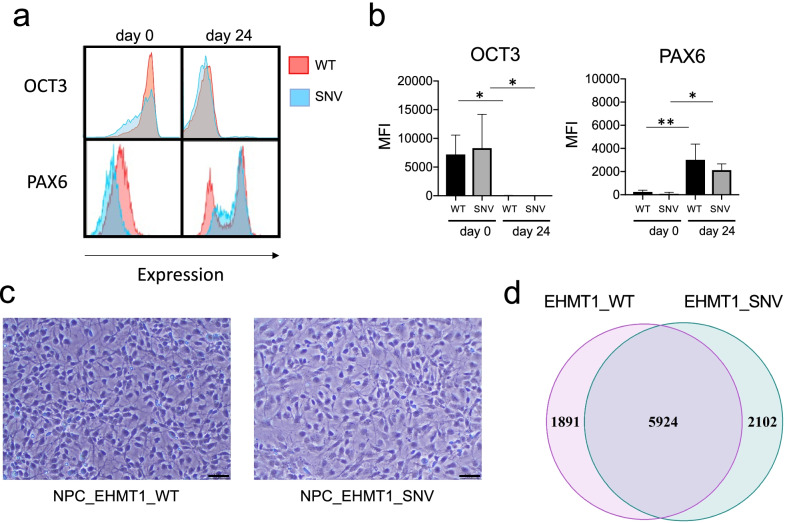
*EHMT1*_WT and *EHMT1*_SNV neural cell differentiation. Cells were subject to neural progenitor cell differentiation with cells harvested at day 0 and day 24 for flow cytometry analysis. **a** Histogram plots indicate cellular staining for pluripotent stem cell marker, OCT3, and neural marker, PAX6. **b** Bar graphs indicate mean fluorescence intensity (MFI) for OCT3 and PAX6. (n = 3 experiments with paired WT and SNV clones. *p ≤ 0.05, **p ≤ 0.01, Kruskal–Wallis, Two stage linear set-up of Benjamini, Krieger and Yekutieli). **c** NPC light microscopy images, black bar indicates 100 micron. **d** Venn diagram indicates number of DEGs for *EHMT1*_WT and *EHMT1*_SNV during iPSC neural differentiation

The *EHMT1*_SNV, and *EHMT1*_WT, iPSCs were induced for neural differentiation to form neural progenitor cells (NPCs). Principal component analysis (PCA) and hierarchical clustering of the normalised RNA-seq data (Additional file 1: Fig. S1b, c) indicated clear separation between *EHMT1*_SNV and *EHMT1*_WT cells. In keeping with expected changes in gene expression for neuronal cell differentiation we determined decreased expression of the OCT3/4 stem cell marker, with a significant increase in expression of the neural cell marker PAX6 by flow cytometry analysis (Fig. 2a, b), and this was similar in the *EHMT1*_WT and *EHMT1*_SNV, iPSC and NPCs, respectively. In addition, NPCs with radial alignment and bipolar morphology for the *EHMT1*_WT and *EHMT1*_SNV NPCs were visually determined (Fig. 2c). At the RNA expression level *EHMT1* showed reduced expression in *EHTM1_*SNV versus *EHMT1*_WT NPCs, however, did not meet our fold change threshold for DEGs (log_2_FC =  − 0.47, *p* = 0.0034).

DEG analysis for the iPSCs compared to NPCs confirmed neural differentiation for *EHMT1*_WT and *EHMT1*_SNV cells. There were 7815 DEGs in *EHMT1*_WT iPS cells during differentiation, and 8026 DEGs in *EHMT1*_SNV cells, with 5924 DEGs common to both (Fig. 2d). We performed gene set enrichment analysis on the DEGs using the EnrichR software. We compared our DEGs against gene sets associated with tissues (ARCHS4_Tissues), and cell function (GO_Biological Process). ARCHS4_Tissues indicated significant enrichment for the *EHMT1*_WT iPS cell to NPC DEGs during neuronal differentiation in the following top five genesets associated with nerve and brain: motor neuron (*p* = 1.14 × 10^−18^), spinal cord (*p* = 1.47 × 10^−14^), spinal cord-bulk (*p* = 1.47 × 10^−14^), Brain bulk (*p* = 2.10 × 10^−12^), and superior frontal gyrus (*p* = 8.79 × 10^−12^). Similarly, for the *EHMT1*_SNV iPS cell to NPC DEGs, the top enriched ARCHS4_Tissues genesets were: motor neuron (*p* = 4.33 × 10^−37^), spinal cord (*p* = 9.61 × 10^−32^), spinal cord-bulk (*p* = 9.61 × 10^−32^), Superior frontal gyrus (*p* = 7.03 × 10^−22^), and brain-bulk (*p* = 1.84 × 10^−20^). Furthermore, the top enriched GO_Biological_Process for both *EHMT1*_WT and *EHMT1*_SNV was nervous system development (*p* = 0.00412 and *p* = 1.17 × 10^−4^, respectively).

This data indicated the capacity for *EHMT1*_WT and *EHMT1*_SNV iPSCs to differentiate into NPCs.

### *EHMT1*_SNV differences in neural differentiation reflect Kleefstra syndrome

**Fig. 3:**
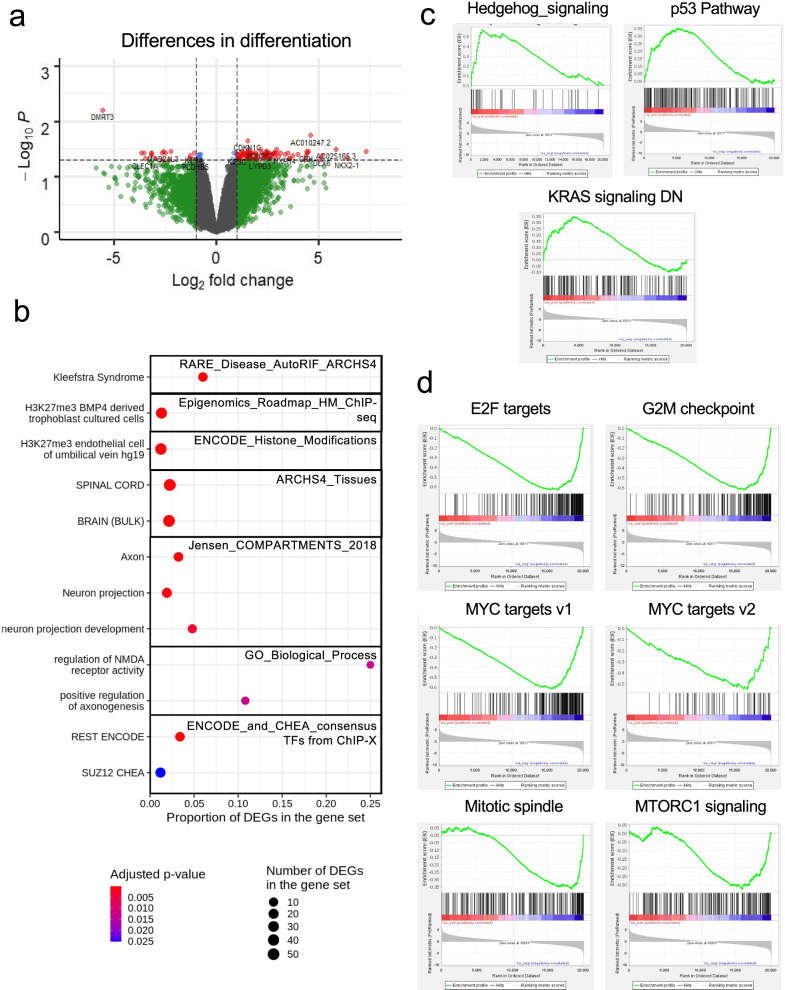
Differences in gene expression during differentiation for *EHMT1_WT* and *EHMT1_SNV* cells*.* iPSC clones for *EHMT1*_WT and *EHMT1*_SNV, were differentiated to NPCs and DEGs determined for the differences in expression during differentiation. **a** Volcano plot for *EHMT1*_SNV compared to *EHMT1*_WT for the differences in expression during differentiation. DEGs are shown in red. Grey dot adjusted *p* ≥ 0.05 and |log_2_FC|≤ 1; green dot adjusted *p* ≥ 0.05 and |log_2_FC|> 1; blue dot adjusted *p* < 0.05 and |log_2_FC|≤ 1; red dot adjusted *p* < 0.05 and |log_2_FC|> 1. **b** Dot plots of significantly enriched gene sets associated with differences in expression during differentiation identified with Enrichr. **c** Enrichment plots for GSEA hallmark significant gene sets for genes showing upregulation during differentiation for *EHMT1*_SNV compared to *EHMT1*_WT. **d** Enrichment plots for GSEA hallmark significant gene sets for genes showing downregulation during differentiation for *EHMT1*_SNV compared to *EHMT1*_WT

Next, we determined the genes that change expression during neural differentiation that are different between the *EHMT1*_SNV and *EHMT1*_WT cells (ie. [NPC_SNV – iPSC_SNV] – [NPC_WT – iPSC_WT]; Fig. 3a). We determined 109 significant DEGs, including 89 genes with increased expression, 19 genes with decreased expression, and 58 genes that were brain and/or neuronal related. The top up-regulated protein coding gene was the homeobox transcription factor NKX2.1, which is involved in establishment of a permissive chromatin state for the formation of medial ganglionic eminence progenitors and production of GABAergic neurons [37]. The top down-regulated gene was DMRT3. The loss of this gene induces massive production of GABAergic neurons [38].

Enrichr analysis of the DEGs indicated enrichment in genesets associated with methylation, cell type, tissue type, cell compartment, molecular function, cellular process, pathways, and transcription factors (Additional file 2: Table S3. Enrichr Diff in Diffn EHMT1_SNV v EHMT1_WT), with identification of the rare disease Kleefstra Syndrome (Fig. 3b).

For Epigenomics_Roadmap_HM_ChIP-seq gene sets significant enrichment was seen for H3K27me3 BMP4 derived trophoblast cultured cells (*p* = 9.29 × 10^−5^). Further ENCODE_Histone_modifications_2015 top 5 enriched gene sets relevant to human were: H3K27me3 endothelial cell of umbilical vein hg19 (*p* = 1.62 × 10^−5^), H3K27me3 BJ hg19 (*p* = 1.62 × 10^−5^), H3K27me3 kidney epithelial cell hg19 (*p* = 1.62 × 10^−5^), H3K27me3 cardiac mesoderm hg19 (*p* = 2.06 × 10^−4^), and H3K27me3 bronchial epithelial cell hg19 (*p* = 0.0023). This data indicated changes in histone methylation due to the effect of *EHTM1*_SNV on neural cell differentiation.

Enrichment in tissue and cell associated gene sets were next examined. The top 5 enriched ARCHS4_Tissues gene sets were: spinal cord (*p* = 2.05 × 10^−19^), spinal cord-bulk (*p* = 2.05 × 10^−19^), brain-bulk (*p* = 5.04 × 10^−18^), superior frontal gyrus (*p* = 5.04 × 10^−18^), and cingulate gyrus (*p* = 1.42 × 10^−16^), with the same top 5 gene sets for the overexpressed DEGs. In Jensen_Compartments the top five significant gene sets were: Axon (*p* = 1.6 × 10^−4^), cell projection (*p* = 5.05 × 10^−4^), Neuron projection (*p* = 5.05 × 10^−4^), somatodendritic compartment (*p* = 0.0011), and site of polarized growth (*p* = 0.0011). For GO_Biological_Process_2018 significantly enriched gene sets from all DEGs were: neuron projection development (*p* = 0.0028), regulation of N-methyl-D-aspartate receptors (NMDAR) activity (*p* = 0.0126), positive regulation of axogenesis (*p* = 0.0126), and positive regulation of axon extension (*p* = 0.0284). Interestingly, GO_Biological_Process enriched gene sets for overexpressed DEGs analysis identified the additional genesets: negative regulation of transferase activity (*p* = 0.04365), regulation of glutamate receptor signalling pathway (*p* = 0.0436), regulation of neurotransmitter receptor activity (*p* = 0.0436), regulation of axon extension (*p* = 0.0436), and regulation of filopodium assembly (*p* = 0.0460). This data indicated changes in neural differentiation that affect cell specification and cell function.

Next, we investigated enriched transcription factor and hub proteins. Significantly enriched gene sets in ENCODE_and_CHEA_Consensus_TFs_from_ChIP-X were: REST ENCODE (*p* = 1.21 × 10^−5^) and SUZ12 CHEA (*p* = 0.0252). REST, RE1 Silencing Transcription Factor, is a neural regulator gene that exhibits both positive and negative gene regulation dependent on the stage of cell development [39]. SUZ12 is part of the polycomb repressive complex 2 (PRC2) which is an epigenetic regulator with H3K27me3 histone methyltransferase activity in chromatin modification associated with repression of cell proliferation or self-renewal genes [40].

Strikingly, in investigation of enriched gene sets associated with disease (Enrichr Rare Disease_AutoRIF_ARCHS4_predictions) the Kleefstra Syndrome geneset was significantly enriched (*p* = 3.61 × 10^−8^).

To further characterise the changes in gene expression for the difference in differentiation for *EHMT1*_SNV compared to *EHMT1*_WT neural cell differentiation, we performed GSEA using the DEGs from the ranked gene list (Fig. 3c and d; Suppl Table 4. GSEA Diff in Diffn *EHMT1*_SNV v *EHMT1*_WT). Significantly enriched hallmark gene sets associated with overexpressed genes included: Hedgehog signalling, myogenesis, p53 pathway, and KRAS signalling DN. Hallmark gene sets associated with underexpressed genes included: E2F targets, G2M checkpoint, MYC targets v1, MYC targets v2, mitotic spindle, MTORC1 signalling, epithelial mesenchymal transition, and androgen_response. Relevant to neural cell differentiation is the Hedgehog signalling which is regulated by REST [41] and involved in GABAergic neuron formation, and KRAS (DN) which suppresses neuronal cell differentiation from iPSCs [42]. Further, *EHMT1*_SNV neural cell differentiation had reduced cell division pathway gene expression for E2F, G2M, mitotic spindle, and MYC; with increased p53 pathway that suppresses cell proliferation.

These data indicate that neural cell differentiation of *EHMT1*_SNV iPSCs, compared to *EHMT1*_WT iPSCs, had increased neural gene expression, with increased GABAergic related genes and pathways, as well as repressed cell proliferation/cell division. The genes associated with these differences were enriched for genes associated with Kleefstra Syndrome.

### Changes in *EHMT1*_SNV transcripts and transcription factors identifies key regulators REST and SP1

We performed upstream regulatory analysis to identify transcription factors (TFs) driving the observed changes in gene expression. We selected the 500 bp upstream region of annotated gene promoters of differentially expressed genes to search for motifs using MEME/*TomTom*, DREME/*TomTom*, and AME to identify key regulatory TFs (Additional file 2: Table S5. Transcription factors Diff in Diffn_MEME DREME AME). MEME/*TomTom* identifies de novo enriched transcription factors, DREME identifies differential motifs, and AME identifies known transcription factors.

In both the *EHMT1*_WT, and *EHMT1*_SNV, iPSC to NPC neural cell differentiation analysis of downregulated DEGs, MEME discovered significant overrepresentation of KLF4 one of the four key Yamanaka Factors for maintaining stem cell integrity, *p* = 0.00047 and *p* = 0.0183, respectively [43]. Similarly, in AME analysis of underexpressed DEGs for the *EHMT1*_WT, and *EHMT1*_SNV, iPSC to NPC neural cell differentiation there was significant overrepresentation of KLF4, *p* = 1.14 × 10^−9^, and *p* = 0.00047, respectively [43]. This confirmed a downregulation of the KLF4 transcription factor as expected in neural cell differentiation.

For genes that show differences in expression during differentiation for *EHMT1*_SNV versus *EHMT1*_WT, MEME analysis of overexpressed genes identified significant transcription factors were ZNF263, EGR1, SP1, and SP2. In MEME analysis of down-regulated genes there were no significantly enriched TF binding sites identified.

ZNF263 is a chromatin binding zinc finger protein involved in chromatin loop formation and is capable of recruiting DNMT co-repressor complexes to silence transcription via H3K27me3 modification [44]. In rodents, EGR1 (Early growth response 1) co-localizes with Nestin expression and is a marker of NPCs [45]. SP1 regulates cell cycle, and in concert with E2F3 controls cell cycle transcription networks and epigenetic modifiers that determines cell fate [46, 47]. SP2 is a regulator of late neurogenic gene expression [48].

**Fig. 4:**
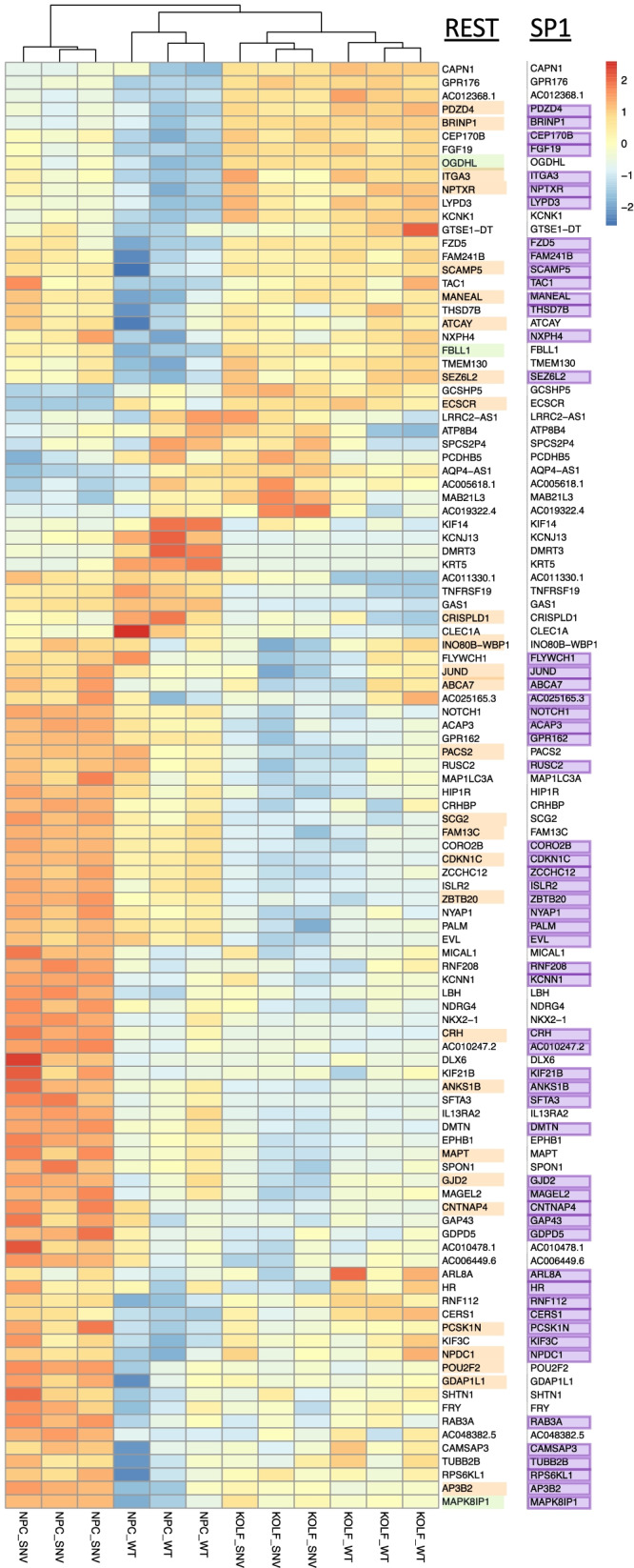
Genes showing differences in expression during differentiation for *EHMT1*_SNV compared to *EHMT1*_WT reveal predominant REST and SP1 modulation. Heatmap of normalised log_2_ counts per million, where genes have been centred and scaled by the standard deviation for the 109 genes showing differences in expression during differentiation for *EHMT1*_SNV compared to *EHMT1*_WT. Genes with REST and SP1 motifs are highlighted. List of genes that were identified with REST motif (green highlight) or in REST gene sets (pale orange highlight), and with SP1 motif (purple highlight)

AME analysis of up-regulated genes showed enrichment for binding of REST, a regulator of neural differentiation [39], and SP1, involved in cell cycle regulation [46]. Previous studies indicate that the REST TF binds CYDL and recruits EHMT1 to the complex to repress gene transcription [49]. The REST TF motif was discovered upstream of three DEGs: MAPK8IP1, OGDHL and FBLL1. We also examined REST genesets from other sources (ENCODE_and_CHEA_Consensus _TFs_from_ChIP-X and ENCODE_TF_ChIP_seq_2015) and determined a further 28 REST related genes (Fig. 4). This indicates that 31 of the 109 DEGs could be directly regulated by REST. For the AME analysis of up-regulated genes 57 of the 109 DEGs were identified with an SP1 transcription factor motif (Fig. 4).

We further investigated whether there were differences at the level of individual transcripts by performing differential transcript expression (DTE) analysis (Suppl Table 6. DTE Diff of Diffn). Interestingly, however, in the DTE for *EHMT1*_WT NPCs versus iPSCs there was a significant change in the expression of transcript SP1-204 (log_2_FC = 1.41, *p* = 0.03). This shorter SP1-204 variant, of 162 amino acids, is a stronger activator of transcription than SP1-201 [50], where higher SP1-204 transcripts levels are found in G phase cells compare to S phase cells. This indicates increased SP1-204 expression in the EHMT1_WT cells may contribute to changes in gene expression relevant to cell cycle and neural cell differentiation.

## Discussion

Rare disease diagnosis is often difficult and determining the relevance of genetic variants of uncertain significance leads to significant delays in patient diagnosis. In this study a patient with a genetic variant in the *EHMT1* gene is assessed with relevance to Kleefstra Syndrome [36]. CRISPR SNV gene editing in KOLF2 iPSCs, neuronal disease modelling, and functional genomics identified Kleefstra disease and associated changes in neural differentiation. Importantly, the study combination of CRISPR with click chemistry and high throughput genetic variant screening methodology enabled the rapid identification of cells containing the genetic variant. In addition, the RNA-seq expression data confirms successful neural cell differentiation of *EHMT1*_SNV iPSCs, including relevant neural expression markers and transcription factors. However, despite successful differentiation, changes in cell function, epigenetics, neurotransmitters, cell proliferation, and neural differentiation were identified in the *EHMT1*_SNV, Kleefstra-like cells. In addition, the study linked transcription factors REST and SP1 to disease specific changes relevant to Kleefstra Syndrome that portend pivotal cellular changes underpinning disease mechanism.

The study of rare genetic diseases identifies single nucleotide variants in the genome that may or may not be disease causative. Current methods for the rapid introduction of these genetic variants in cells is poor. Whilst CRISPR gene editing for knock out genes has been robustly developed, the introduction of single nucleotide changes in DNA requires HDR which occurs at frequencies of < 1%. In this study we employed CRISPR click chemistry and amplicon sequencing to identify iPSC clones that were heterozygous for the EHMT1_c.3430C > T (p.Gln1144*) patient variant. Historically iPSCs have been difficult to edit with CRISPR however our methodology rapidly identified functional iPSCs that were stimulated for neuronal modelling to determine cellular changes relevant to Kleefstra Syndrome and identify novel disease mechanisms.

Importantly, when examining the genes that show differences in expression during differentiation for *EHMT1*_SNV cells compared to *EHMT1*_WT cells, findings were consistent with the patient disease phenotype, Kleefstra Syndrome, were identified. In this study there were no observed differences in neural marker expression and cell morphology between the *EHMT1*_WT cells and *EHMT1*_SNV iPSCs or NPCs. Similarly, others have determined no significant phenotypic differences in neurons derived from patients with Kleefstra Syndrome and healthy controls [51]. We were able to determine reduced EHMT1 gene and protein expression in the *EHMT1*_SNV iPSCs and NPCs in keeping with findings in Kleefstra patient cells [51–53]. In addition, in this study DEG analysis of *EHMT1*_WT and *EHMT1*_SNV iPSC to NPC differentiation indicated similar neural differentiation profiles. Strikingly, however DEG analysis that tested for differences in gene expression during differentiation for *EHTM1*_SNV compared to *EHMT1*_WT revealed distinct changes related to histone methylation, cell proliferation/cell cycling, and NPC differentiation/maintenance.

Epigenetic modification of chromatin in neurons has a role in cognitive impairment and intellectual disability [54] and EHMT1 is long established in H3K9me1 and me2, for heterochromatin formation and gene repression [5, 6]. Dysregulation of H3K9me1/me2 is thought to lead to alternative histone methylation by allowing access of other methyl transferases. Genes with significantly different changes in expression during differentiation when comparing *EHMT1*_SNV to *EHMT1*_WT were associated with strong dysregulation of H3K27 methylation. Interestingly, we observed TFs involved in H3K27me3 including EZH2 methyltransferase [55], and ZNF263 via SIX3 modulation [44]. Reportedly, ZNF263 also plays a role in chromatin restructuring via the CCCTC-binding factor (CTCF) chromatin loop formation [44].

Dysregulation of cell cycle length has been indicated as a driver of premature differentiation of neural progenitor cells [46], and siRNA EHMT1 knockdown experiments indicate reduced cell proliferation and increased cell differentiation [56]. Furthermore, TFs play a crucial role in control of the cell cycle [57], here, in *EHMT1*_SNV neural differentiation we find decreased activity of E2F, and SP1, with increased activity of the cell proliferation regulator p53. We also identified reduced cellular proliferation profiles in *EHMT1*_SNV during differentiation via reduced expression of genes involved in G2/M [58], mTORC1 pathways, and KRAS signalling [42] and conversely increased expression of genes involved in Hedgehog signalling [41].

Functional genomic analysis of *EHMT1*_SNV neural differentiation identified increased neurotransmitter receptor activity. Similarly, others identified differences in action potential decay, with lower frequency network bursts, with longer burst and inter-burst duration in Kleefstra Syndrome patient derived cells [51]. We demonstrate a further change in *EHMT1*_SNV neural differentiation indicative of increased GABAergic neurons via downregulation of DMRT3. Similarly, others observed increased GABAergic neurons in DMRT3 knockout experiments in mice [38], whilst in  *Ehmt1* ^±^ mice dysregulation of GABAergic interneurons reportedly leads to increased neuron firing [51]. Further, *EHMT1*_SNV overexpression of NKX2.1 was observed, where reportedly NKX2.1 establishes a permissive chromatin state for the production of GABAergic interneurons [37].

Neural differentiation of *EHMT1*_SNV iPSC cells indicated a change in both stem cell and neural associated transcription factors including MYC, REST and SP1. A negative association with the MYC targets likely indicates loss of stemness in these cells as they are induced for neural cell differentiation [59, 60].

REST regulates neural cell differentiation and governs neuronal cell phenotype and specification [39]. The REST transcription factor forms a complex with EHMT1 via its interaction with CDYL [49], for histone methylation and chromatin modulation in the regulation of gene expression. Accordingly, during neural cell differentiation a consequence of the EHMT1 truncated protein may be disruption of the REST/EHMT1 complex, which leads to the perturbations in gene expression observed in Kleefstra Syndrome. Potentially REST binds DNA yet is unable to effectively recruit EHMT1 p.Gln1144* protein, or EHMT1 p.Gln1144* is recruited but does not function.

The TF motif analysis further identified SP1 as being associated with genes showing differences in expression during differentiation for *EHMT1*_SNV versus *EHMT1*_WT. We also identified differential expression of a short SP1-204 transcript during EHMT_WT iPSC differentiation that is a strong activator of cell cycle gene expression. Potentially loss of SP1-204 expression in *EHMT1*_SNV cells contributes to decreased cell cycle activity and altered gene expression. Alternatively, SP1 recruitment to the OCT4 promoter, via PML (promyelocytic leukemia 1), maintains open chromatin for OCT4 gene expression in stem cells [61], where induction of neural differentiation leads to SP1 dissociation and subsequent recruitment of an EHMT2 complex leading to heterochromatin formation and gene suppression [61]. We observed similar downregulation of OCT4 (POU5F1) in neural differentiation for both the *EHMT1*_SNV and *EHMT1*_WT iPSCs, potentially indicating that in this scenario EHMT2 works as a homodimer, or the EHMT1/EHMT2 complex remains functional in this capacity. In other work, in viral infection SP1 regulates HIST1H1C which subsequently modulates EHMT1/EHMT2 complex formation and gene expression [62]. Here the genes showing differences in expression during differentiation for *EHMT1*_SNV compared to *EHMT1*_WT similarly provide a link between SP1 and EHMT1/EHTM2 in the regulation of gene expression.

Interestingly there is interplay between SP1 and REST gene expression in adult neurons where REST binding to chromatin can suppress SP1 gene expression, alternatively, in other neural states/conditions SP1 has been shown to positively modulate REST expression [63]. Additionally, in neural cells SP1 directly binds the promoter of gene SYN1 (Synapsin I) leading to gene expression, whilst REST binding of the SYN1 promoter suppresses gene expression [64]. SYN1 plays a crucial role in neurogenesis, including axonogenesis and synaptogenesis, and has a role in synaptic transmission and mature neuron plasticity [64]. Here, we demonstrate that REST and SP1 interplay to co-ordinately regulate gene expression of a specific subset of genes associated with Kleefstra Syndrome.

The use of bioinformatics in this study has highlighted significant pathways relevant to Kleefstra syndrome. A caveat of the bioinformatics methods is the reliance on available genesets to determine changes in cell function. As more genetic variants in disease are identified and molecular pathways delineated these genesets will however become more expansive.

## Conclusions

CRISPR edited *EHMT1*_SNV iPSC with neural disease modelling and functional genomics analysis clearly validated the patient genetic variant in Kleefstra syndrome. EHMT1 haploinsufficiency, a hallmark of Kleefstra syndrome, was confirmed in the *EHMT1*_SNV cells by decreased EHMT1 protein expression. Many genetic diseases do not have a specific functional test and this study argues for the utility of patient genetic variant analysis with our CRISPR HDR gene editing, disease modelling and functional genomics analysis pipeline. In addition, transcription factors, REST and SP1, key to the etiology of Kleefstra syndrome were identified providing insight into disease mechanism which may facilitate the identification of drug targets for treatment. Importantly, the pipeline can be modified to assess genetic variants relevant to many genes relevant to rare, or other, disease as iPSCs can be modelled to neuronal, cardiac, lung or other tissue types.

## Supplementary Information

**Additional file 1: Figure S1**. Differential gene expression in iPS cells and NPCs with *EHMT1_WT* and *EHMT1_SNV.* **a**, schematic of CRISPR gene edit in EHMT1. C > T mutation indicated in yellow and capital letters indicate silent mutations. The iPS cell clones for *EHMT1*_WT and *EHMT1*_SNV were differentiated to NPCs EHMT1_WT, and *EHMT1*_SNV. **b** Principal component analysis. **c** Euclidean distance between samples

**Additional file 2: Table S1**. EHMT1 off target.** Table S2**. HPO Terms for patient. **Table S3**. Enrichr Diff in Diffn *EHMT1*_SNV v *EHMT1*_WT. **Table S4**. GSEA Diff in Diffn Suppl Tables_*EHMT1*_SNV v *EHMT1*_WT. **Table S5**. Transcription factor tables Diff in Diffn_AME DREME MEME. **Table S6**. DTE Diff in Diffn.

## Data Availability

Raw FASTQ files and processed count data are available at the Gene Expression Omnibus repository under accession number GSE178646.

## Abbreviations

CRISPR: Clustered regularly interspaced short palindromic repeats
crRNA: Cripsr RNA
DEG: Differentially expressed gene
DTE: Differentially expressed transcript
gDNA: Genomic deoxyribonucleic acid
HDR: Homology directed repair
iPSC: Inducible pluripotent stem cells
NGS: Next generation sequencing
NPC: Neural progenitor cell
PAM: Protospacer adjacent motif
RNA: Ribonucleic acid
SNV: Single nucleotide variant
ssDNA: Single stranded DNA
TF: Transcription factor
tracrRNA: Trans-acting crRNA
WT: Wildtype
VUS: Variant of uncertain significance
