# Supplementary figures and images for: CRISPR single base editing, neuronal disease modelling and functional genomics for genetic variant analysis: pipeline validation using Kleefstra syndrome EHMT1 haploinsufficiency
Source: Stem Cell Res Ther. 2022 Feb 9;13:69. doi: 10.1186/s13287-022-02740-3 (PMC8827184; doi:10.1186/s13287-022-02740-3)

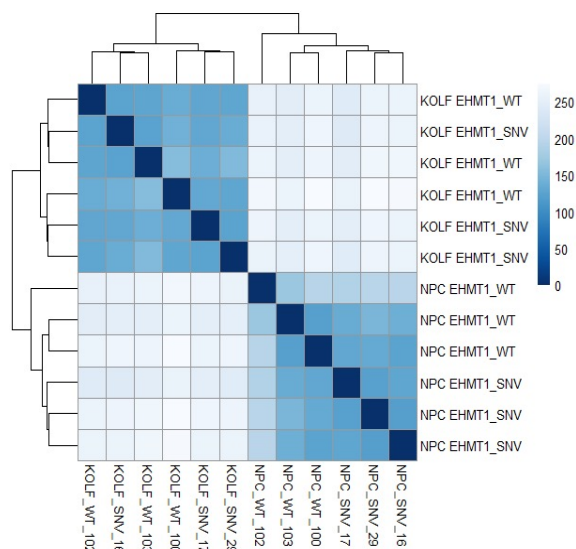

Supplement: Supplementary file 1 — Additional file 1: Figure S1. Differential gene expression in iPS cells and NPCs with EHMT1_WT and EHMT1_SNV. a, schematic of CRISPR gene edit in EHMT1. C > T mutation indicated in yellow and capital letters indicate silent mutations. The iPS cell clones for EHMT1_WT and EHMT1_SNV were differentiated to NPCs EHMT1_WT, and EHMT1_SNV. b Principal component analysis. c Euclidean distance between samples [file 13287_2022_2740_MOESM1_ESM.pdf]
