# Supplementary material for: CRISPR single base editing, neuronal disease modelling and functional genomics for genetic variant analysis: pipeline validation using Kleefstra syndrome EHMT1 haploinsufficiency
Source: Stem Cell Res Ther. 2022 Feb 9;13:69. doi: 10.1186/s13287-022-02740-3 (PMC8827184; doi:10.1186/s13287-022-02740-3)
Supplement: Supplementary file 2 — Additional file 2: Table S1. EHMT1 off target. Table S2. HPO Terms for patient. Table S3. Enrichr Diff in Diffn EHMT1_SNV v EHMT1_WT. Table S4. GSEA Diff in Diffn Suppl Tables_EHMT1_SNV v EHMT1_WT. Table S5. Transcription factor tables Diff in Diffn_AME DREME MEME. Table S6. DTE Diff in Diffn. [file 13287_2022_2740_MOESM2_ESM.docx]

**SUPPLEMENTARY DATA**

**Supplementary Figure 1. Differential gene expression in iPS cell and NPCs with *EHMT1_WT* and *EHMT1_SNV.*** a, schematic of CRISPR gene edit in EHMT1. C>T mutation indicated in yellow and capital letters indicate silent mutations. The iPSC clones for EHMT1_WT and EHMT1_SNV were differentiated to NPCs EHMT1_WT, and EHMT_SNV. b, Principal component analysis. c, Euclidean distance between samples.

**Supplementary Table 1. EHMT1 off target.**

| **Sequence** | **PAM** | **Gene** | **Locus** | **F1** | **R1** |
| --- | --- | --- | --- | --- | --- |
| CACCTGCGGTACATCCCACC | CAG | UBE2J2 | chr1:  +1254114 | CACAGACACAAGCAGCATGA | GCTGGCCTTCACAGTAGGTAA |
| TCCCACAAGGACATCCCACC | TGG |  | chr18:  -79770525 | GGTCTCTGCTGTGAGGAGGA | TCCTCAAATGCTCGGACTG |
| ACCCTTCAGGACATCCCACC | AAG |  | chr18:  +11553299 | ATCAGCGGTGGCTACCATAC | ATCCAGAAGTGGCTGCAAGT |
| TCGCTGCGGGGCATCCCACC | GAG | GPR146 | chr7:  -1055051 | CGCTCGACAGTAGACCCTTC | AGAGCTCTGGTGAGGACCTG |
| GCCTTGCCGGGCATCCCACC | AAG |  | chrX:  +12601831 | TCCAACTCCCATCTTCGTTC | TCAGGGCTCAAAGCAAAAGT |
| GCCCTGC-TGACATCCCACC | AGG |  | chr15:  +78804497 | CTGCAGTTCCCAGGCTCTAA | CCAGCCTACCCAGTCTCTTG |

**Supplementary Table 2. HPO Terms for patient.**

| **Supplementary Table 1: HPO terms for patient** |
| --- |
| HP:0001513 Obesity |
| HP:0002205 Recurrent respiratory infections |
| HP:0010804 Tented upper lip vermilion |
| HP:0000028 Cryptorchidism NOT |
| HP:0001252 Muscular hypotonia |
| HP:0004414 Abnormality of the pulmonary artery |
| HP:0000391 Thickened helices |
| HP:0000303 Mandibular prognathia |
| HP:0000272 Malar flattening |
| HP:0011800 Midface retrusion |
| HP:0000316 Hypertelorism |
| HP:0000365 Hearing impairment |
| HP:0004322 Short stature |
| HP:0002553 Highly arched eyebrow |
| HP:0000687 Widely spaced teeth |
| HP:0001773 Short foot |
| HP:0000508 Ptosis |
| HP:0000407 Sensorineural hearing impairment |
| HP:0002558 Supernumerary nipple |
| HP:0001807 Ridged nail |
| HP:0001344 Absent speech |
| HP:0001642 Pulmonic stenosis |
| HP:0000307 Pointed chin |
| HP:0000691 Microdontia |
| HP:0000252 Microcephaly |
| HP:0001263 Global developmental delay |
| HP:0000463 Anteverted nares |
| HP:0001156 Brachydactyly syndrome |
| HP:0012743 Abdominal obesity |
| HP:0200006 Slanting of the palpebral fissure |
| HP:0000664 Synophrys |
| HP:0009623 Proximal placement of thumb |

**Supplementary Table 3. Enrichr Diff in Diffn EHMT1_SNV v EHMT1_WT.**

| **Epigenomics** | | | |
| --- | --- | --- | --- |
| Term | Overlap | P-value | Adjusted P-value |
| H3K27me3 H1 BMP4 Derived Trophoblast Cultured Cells | 35/2683 | 3.01E-07 | 9.29E-05 |
| **ENCODE_Histone** | | | |
| Term | Overlap | P-value | Adjusted P-value |
| H3K27me3 endothelial cell of umbilical vein hg19 | 42/3393 | 4.66E-08 | 1.62E-05 |
| H3K27me3 BJ hg19 | 31/2090 | 1.10E-07 | 1.62E-05 |
| H3K27me3 kidney epithelial cell hg19 | 32/2217 | 1.20E-07 | 1.62E-05 |
| H3K27me3 small intestine mm9 | 28/2000 | 1.75E-06 | 1.76E-04 |
| H3K27me3 cardiac mesoderm hg19 | 39/3485 | 2.56E-06 | 2.06E-04 |
| H3K27me3 bronchial epithelial cell hg19 | 26/2082 | 3.44E-05 | 0.00231357 |
| H3K4me1 brown adipose tissue mm9 | 25/2000 | 4.99E-05 | 0.00287348 |
| H3K27me3 kidney mm9 | 22/1675 | 7.57E-05 | 0.00381181 |
| H3K27me3 GM12878 hg19 | 33/3221 | 1.29E-04 | 0.00576702 |
| H3K27me3 mammary epithelial cell hg19 | 28/2586 | 1.99E-04 | 0.00802801 |
| H3K27me3 liver mm9 | 23/2000 | 3.65E-04 | 0.01224608 |
| H3K27me3 testis mm9 | 23/2000 | 3.65E-04 | 0.01224608 |
| H3K27me3 CD14-positive monocyte hg19 | 33/3445 | 4.62E-04 | 0.01431531 |
| H3K27me3 A549 hg19 | 29/2891 | 5.46E-04 | 0.01571871 |
| H3K27me3 heart mm9 | 22/2000 | 9.13E-04 | 0.02452385 |
| H3K27me3 fibroblast of lung hg19 | 29/3052 | 0.00131491 | 0.03311921 |
| **ARCHS4_Tissues** | | | |
| Term | Overlap | P-value | Adjusted P-value |
| SPINAL CORD | 52/2316 | 3.83E-21 | 2.05E-19 |
| SPINAL CORD (BULK) | 52/2316 | 3.83E-21 | 2.05E-19 |
| BRAIN (BULK) | 50/2316 | 1.88E-19 | 5.04E-18 |
| SUPERIOR FRONTAL GYRUS | 50/2316 | 1.88E-19 | 5.04E-18 |
| CINGULATE GYRUS | 48/2316 | 7.98E-18 | 1.42E-16 |
| FETAL BRAIN | 48/2316 | 7.98E-18 | 1.42E-16 |
| MOTOR NEURON | 47/2316 | 4.91E-17 | 7.51E-16 |
| CEREBRAL CORTEX | 46/2316 | 2.91E-16 | 3.89E-15 |
| PREFRONTAL CORTEX | 38/2316 | 1.13E-10 | 1.34E-09 |
| ASTROCYTE | 36/2316 | 1.90E-09 | 2.03E-08 |
| BETA CELL | 35/2316 | 7.32E-09 | 7.12E-08 |
| CEREBELLUM | 34/2316 | 2.71E-08 | 2.23E-07 |
| SENSORY NEURON | 34/2316 | 2.71E-08 | 2.23E-07 |
| DENTATE GRANULE CELL | 33/2316 | 9.64E-08 | 7.37E-07 |
| DORSAL STRIATUM | 32/2316 | 3.28E-07 | 2.19E-06 |
| OLIGODENDROCYTE | 32/2316 | 3.28E-07 | 2.19E-06 |
| RETINA | 29/2316 | 9.99E-06 | 6.29E-05 |
| ADIPOSE (BULK TISSUE) | 27/2316 | 7.78E-05 | 4.16E-04 |
| ALPHA CELL | 27/2316 | 7.78E-05 | 4.16E-04 |
| SUBCUTANEOUS ADIPOSE TISSUE | 27/2316 | 7.78E-05 | 4.16E-04 |
| VALVE | 25/2316 | 5.03E-04 | 0.00256077 |
| CARDIAC MUSCLE FIBER | 24/2316 | 0.00118848 | 0.00552903 |
| RESPIRATORY SMOOTH MUSCLE | 24/2316 | 0.00118848 | 0.00552903 |
| RENAL CORTEX | 23/2316 | 0.00267498 | 0.01192596 |
| VENTRICLE | 22/2316 | 0.00572492 | 0.02450264 |
| BREAST (BULK TISSUE) | 21/2316 | 0.01163884 | 0.04789832 |
| **Jensen_COMPARTMENTS** | | | |
| Term | Overlap | P-value | Adjusted P-value |
| Axon | 13/403 | 2.71E-07 | 1.60E-04 |
| Cell projection part | 18/913 | 1.80E-06 | 5.05E-04 |
| Neuron projection | 18/936 | 2.56E-06 | 5.05E-04 |
| Somatodendritic compartment | 14/642 | 9.27E-06 | 0.00112441 |
| Site of polarized growth | 7/137 | 9.51E-06 | 0.00112441 |
| Dendrite | 11/457 | 3.70E-05 | 0.0036457 |
| Neuron part | 19/1287 | 5.49E-05 | 0.00463812 |
| Growth cone | 6/132 | 8.13E-05 | 0.00535002 |
| Filopodium membrane | 3/16 | 8.15E-05 | 0.00535002 |
| Cell projection | 22/1774 | 1.75E-04 | 0.01031609 |
| C-fiber | 6/177 | 4.01E-04 | 0.02104303 |
| Varicosity | 2/6 | 4.27E-04 | 0.02104303 |
| Excitatory synapse | 6/190 | 5.83E-04 | 0.02476595 |
| clathrin-coated vesicle membrane | 4/72 | 6.24E-04 | 0.02476595 |
| Synapse | 12/737 | 6.29E-04 | 0.02476595 |
| Cell projection membrane | 7/284 | 8.89E-04 | 0.03283796 |
| clathrin-coated vesicle | 5/142 | 0.00104301 | 0.03625977 |
| Climbing fiber | 5/150 | 0.0013311 | 0.04370434 |
| Filopodium | 4/90 | 0.00143851 | 0.04474515 |
| Dense core granule | 2/11 | 0.00153925 | 0.04548483 |
| calcium- and calmodulin-dependent protein kinase complex | 5/158 | 0.00167413 | 0.04711478 |
| **GO_Biological Process** | | | |
| Term | Overlap | P-value | Adjusted P-value |
| neuron projection development (GO:0031175) | 8/167 | 3.46E-06 | 0.00282154 |
| regulation of NMDA receptor activity (GO:2000310) | 3/13 | 4.21E-05 | 0.01257594 |
| positive regulation of axonogenesis (GO:0050772) | 4/37 | 4.63E-05 | 0.01257594 |
| positive regulation of axon extension (GO:0045773) | 3/19 | 1.39E-04 | 0.02838709 |
| **ENCODE_CHEA TF** | | | |
| Term | Overlap | P-value | Adjusted P-value |
| REST ENCODE | 13/383 | 1.52E-07 | 1.21E-05 |
| SUZ12 CHEA | 20/1684 | 6.30E-04 | 0.02518777 |

**Supplementary Table 4. GSEA Diff in Diffn Suppl Tables_EHMT1-SNV v EHMT1_WT.**

| **Overexpressed** | | | | |
| --- | --- | --- | --- | --- |
| NAME | SIZE | FDR q-val | RANK AT MAX | LEADING EDGE |
| HALLMARK_HEDGEHOG_SIGNALING | 34 | 0 | 1681 | tags=38%, list=8%, signal=42% |
| HALLMARK_MYOGENESIS | 168 | 4.85E-04 | 4143 | tags=38%, list=21%, signal=48% |
| HALLMARK_PANCREAS_BETA_CELLS | 29 | 0.00580214 | 2852 | tags=48%, list=14%, signal=56% |
| HALLMARK_HYPOXIA | 185 | 0.01220972 | 3276 | tags=28%, list=16%, signal=33% |
| HALLMARK_P53_PATHWAY | 190 | 0.011336 | 5209 | tags=38%, list=26%, signal=51% |
| HALLMARK_HEME_METABOLISM | 170 | 0.01100811 | 4775 | tags=33%, list=24%, signal=43% |
| HALLMARK_KRAS_SIGNALING_DN | 139 | 0.01777458 | 4256 | tags=34%, list=21%, signal=43% |
| HALLMARK_CHOLESTEROL_HOMEOSTASIS | 70 | 0.04812361 | 7362 | tags=56%, list=37%, signal=88% |
| **Underexpressed** | | | | |
| NAME | SIZE | FDR q-val | RANK AT MAX | LEADING EDGE |
| HALLMARK_E2F_TARGETS | 198 | 0 | 3501 | tags=59%, list=17%, signal=70% |
| HALLMARK_G2M_CHECKPOINT | 194 | 0 | 3732 | tags=58%, list=19%, signal=71% |
| HALLMARK_MYC_TARGETS_V1 | 196 | 0 | 4408 | tags=63%, list=22%, signal=80% |
| HALLMARK_MYC_TARGETS_V2 | 58 | 0 | 3291 | tags=36%, list=16%, signal=43% |
| HALLMARK_MITOTIC_SPINDLE | 198 | 9.79E-04 | 1636 | tags=23%, list=8%, signal=25% |
| HALLMARK_MTORC1_SIGNALING | 196 | 0.00518988 | 4115 | tags=33%, list=21%, signal=41% |
| HALLMARK_UNFOLDED_PROTEIN_RESPONSE | 109 | 0.00972723 | 5228 | tags=44%, list=26%, signal=59% |
| HALLMARK_EPITHELIAL_MESENCHYMAL_TRANSITION | 184 | 0.0223285 | 3281 | tags=28%, list=16%, signal=33% |
| HALLMARK_ANDROGEN_RESPONSE | 95 | 0.026769 | 3986 | tags=33%, list=20%, signal=41% |
| HALLMARK_SPERMATOGENESIS | 102 | 0.0284801 | 4288 | tags=37%, list=21%, signal=47% |

**Supplementary Table 5. Transcription factor tables Diff in Diffn_AME DREME MEME.**

| **Upregulated** | | | **Downregulated** | | |
| --- | --- | --- | --- | --- | --- |
| SNV v WT iPSCs | SNV diff-WT diff | SNV v WT NPCs | SNV v WT iPSCs | SNV diff-WT diff | SNV v WT NPCs |
| - | ZNF263 | ZNF384 | ZNF384 | - | ZNF384 |
|  | EGR1 |  |  |  |  |
|  | SP1 |  |  |  |  |
|  | SP2 |  |  |  |  |
|  | SP1 |  |  |  |  |
|  | SPi1 |  |  |  |  |
| **DREME** | | | | | |
| **Up vs background** | | | **Down vs background** | | |
| SNV v WT iPSCs | SNV diff-WT diff | SNV v WT NPCs | SNV v WT iPSCs | SNV diff-WT diff | SNV v WT NPCs |
| - | - | EGR1 | - | - | - |
|  |  | SP2 |  |  |  |
|  |  | EGR2 |  |  |  |
| **AME** | | | | | |
| **Up vs background** | | | **Down vs background** | | |
| SNV v WT iPSCs | SNV diff-WT diff | SNV v WT NPCs | SNV v WT iPSCs | SNV diff-WT diff | SNV v WT NPCs |
| - | REST | ZNF263 | IRF1 | - | ZNF263 |
|  | SP1 | REST |  |  | TCF7L2 |
|  |  | MZF1 |  |  | Klf4 |
|  |  | REST |  |  | SIX2 |
|  |  | ZNF740 |  |  | MIXL1 |
|  |  | EGR2 |  |  | Sox2 |
|  |  | MZF1(var.2) |  |  |  |
|  |  | RREB1 |  |  |  |
|  |  | ESR1 |  |  |  |

**Supplementary Table 6. DTE Diff in Diffn.**

| Ensembl_transcript | Transcript_symbol | Biotype | logFC | AveExpr | adj.P.Val |
| --- | --- | --- | --- | --- | --- |
| ENST00000346798.7 | APP-201 | protein_coding | -9.11753375 | 4.543220601 | 0.000270446 |
| ENST00000636751.2 | TCF4-279 | nonsense_mediated_decay | -4.39609576 | -2.139163286 | 0.022781904 |
| ENST00000644744.1 | KITLG-206 | protein_coding | 3.06964091 | 3.753258927 | 0.003215601 |
| ENST00000572841.1 | SGSM2-204 | retained_intron | 4.0863483 | 0.43907561 | 0.048180891 |
| ENST00000593685.5 | DYRK1B-204 | protein_coding | 4.56439603 | -2.049084391 | 0.014896797 |
| ENST00000449375.1 | RETSAT-205 | protein_coding | 4.57524203 | -2.108809247 | 0.023485053 |
| ENST00000640655.2 | AC116366.3-205 | protein_coding | 4.68817576 | -2.02776081 | 0.047888068 |
| ENST00000381488.10 | ARRB2-203 | protein_coding | 4.73077903 | 0.58290776 | 0.014896797 |
| ENST00000344791.6 | LYSMD4-202 | protein_coding | 4.78567934 | -1.883006044 | 0.017367169 |
| ENST00000282488.11 | MBNL1-202 | protein_coding | 5.20812246 | -1.850921313 | 0.007131643 |
| ENST00000395799.7 | TNRC6A-202 | protein_coding | 5.64267213 | -1.789456598 | 0.003215601 |
| ENST00000435036.6 | MTA1-207 | protein_coding | 5.87446033 | -1.735080239 | 0.007610891 |
| ENST00000534641.5 | GTF2H1-214 | protein_coding | 5.91738801 | 1.065787375 | 0.047888068 |
| ENST00000468747.5 | FAM107B-211 | protein_coding | 8.98613844 | 0.626103107 | 0.017367169 |
| ENST00000451743.6 | MEG3-209 | lincRNA | 10.1405387 | -0.322538026 | 0.001173054 |
| ENST00000430149.3 | CDKN1C-203 | protein_coding | 10.1662895 | -0.374206087 | 0.001173054 |
